# Clinical characteristics and treatment efficacy in patients with primary severe IGF-1 deficiency treated with recombinant IGF-1

**DOI:** 10.3389/fped.2024.1461163

**Published:** 2024-10-28

**Authors:** Dovile Denaite, Ruta Navardauskaite

**Affiliations:** Department of Endocrinology, Medical Academy, Lithuanian University of Health Sciences, Kaunas, Lithuania

**Keywords:** primary severe IGF-1 deficiency, short stature, recombinant IGF-1 analog, mecasermin, side effect

## Abstract

**Aim of the study:**

To evaluate the clinical characteristics and treatment efficacy of patients with severe primary IGF-1 deficiency (PSIGFD) using a recombinant IGF-1 (rhIGF-1).

**Objectives of the study:**

To examine the clinical characteristics of patients with PSIGFD before starting treatment with a rIGF-1. To assess the height changes in patients with PSIGFD, before and after treatment with a rhIGF-1. To analyze the clinical characteristics, side effect frequency, and treatment efficacy with a rhIGF-1 analog in patients with PSIGFD.

**Methods:**

A retrospective analysis was conducted on patients with PSIGFD treated with the rhIGF-1 (mecasermin). Data were collected from patients’ medical records, focusing on the impact of treatment on their growth and monitoring any side effects.

**Results:**

The study showed that treatment with rhIGF-1 positively affects growth rate, especially in the first years of treatment. However, the growth rate decreases over time. The change in height from the beginning to the end of the treatment was 0.76 ± 0.64 SD, with the first quartile at 0.29 SD and the third quartile at 1.14 SD. During the treatment period, patients’ average body mass increased by 0.37 ± 1.35 SD, with the first quartile at −0.33 SD and the third quartile at 0.92 SD. Side effects occurred in 50% of patients, with 40% of patients treated with rhIGF-1 experiencing hypoglycemia during treatment.

**Conclusions:**

Treatment with rhIGF-1 is effective in treating patients with PSIGFD, causing significant improvement in growth, but requires continuous monitoring and treatment adjustment.

## Introduction

Insensitivity to growth hormone (GH), also known as primary insulin-like growth factor 1 (IGF-1) deficiency, is a rare pathological condition that causes significant growth disorders in children, leading to physical and psychological disabilities (1–3). IGF-1 (also known as somatomedin C) is a hormone (a protein composed of 70 amino acids) with a structure very similar to insulin. Its production in the liver is stimulated by GH. IGF-1 promotes systemic body growth and has a growth-promoting effect on almost all body cells, especially skeletal muscles, cartilage, bones, liver, kidneys, nerves, skin, hematopoietic, and lung cells (4, 5).

The diagnosis of primary IGF-1 deficiency (PIGFD) encompasses a wide range of disorders resulting from molecular defects in the GH-IGF-1 axis. These defects can be related to genes encoding proteins that regulate GH binding or signal transmission, as well as IGF-1 synthesis, transport, or action. The location of the defect is associated with different phenotypic manifestations and a spectrum of biochemical anomalies (1, 6–8). The prototype of GH insensitivity, Laron syndrome, usually occurs due to defects in the GH receptor gene or the IGF-1 gene (9). Individuals with this syndrome are characterized by extremely short stature, normal or increased GH secretion, very low IGF-1 concentration, and low IGF-1 response to GH (1, 10).

PIGFD is usually diagnosed in childhood when growth retardation is noticed, and it requires prompt and effective treatment to improve growth and prevent metabolic complications. Recombinant IGF-1 (*mecasermin*) therapy has been shown to be an effective treatment for this condition, improving growth and metabolic parameters. At the start of treatment, a rapid and significant acceleration in growth rate is observed, but the long-term effect of the treatment and individual patient response can vary. In clinical practice, the effectiveness of *mecasermin* treatment is assessed based on changes in height and weight, as well as monitoring for side effects. One of the most common adverse effects is hypoglycemia, which is related to the impact of IGF-1 on glucose metabolism. Therefore, it is especially important to regularly monitor patients’ glucose levels and adjust to each patient's individual reactions during treatment (6, 11).

 However, there is no gold standard or unified clinical criteria for diagnosing primary severe IGF-1 deficiency, which complicates the management and treatment of this condition. This study aims to present cohort data and contribute to the limited global database on the outcomes of recombinant IGF-1 (rIGF-1) treatment. By providing detailed clinical characteristics and treatment outcomes, this research seeks to enhance the understanding of rIGF-1 therapy's efficacy and safety, and address the variability in clinical practice.

## Methods

### Study design

**Figure 1:**
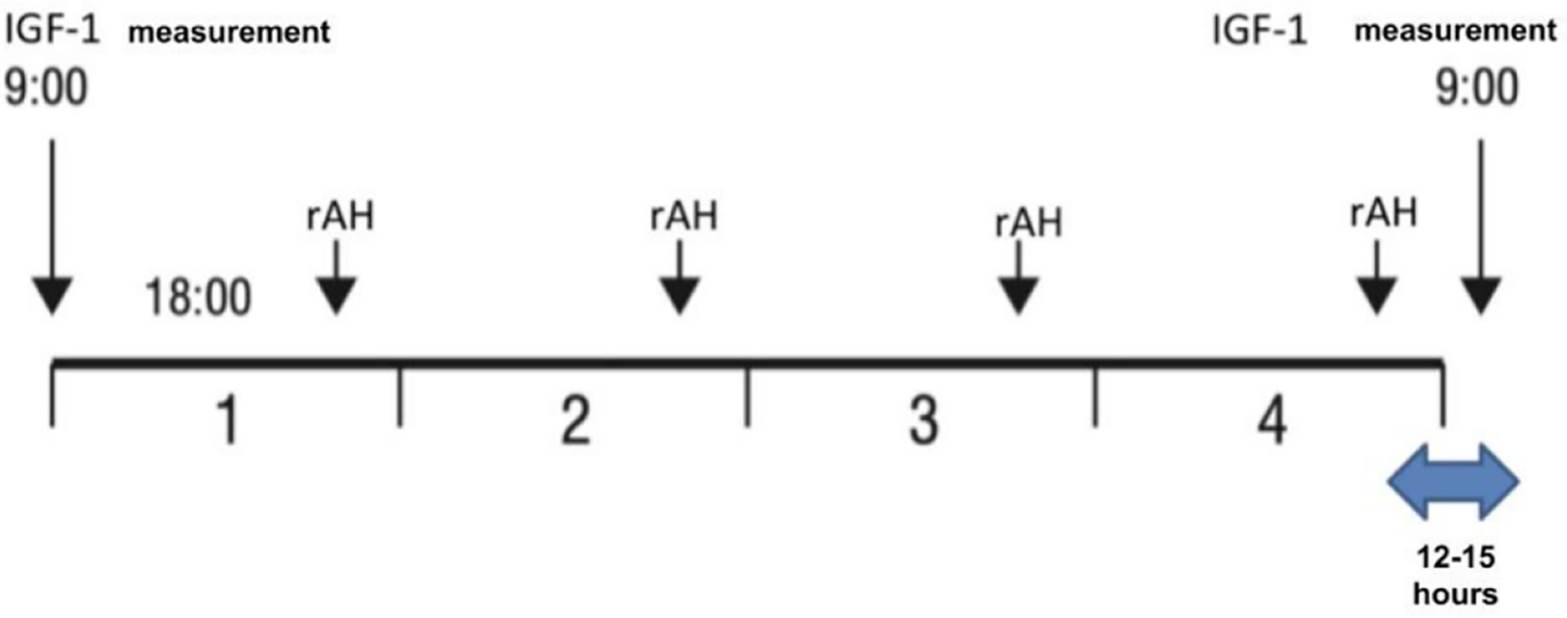
Performance of the IGF-1 generation test.

**Figure 2:**
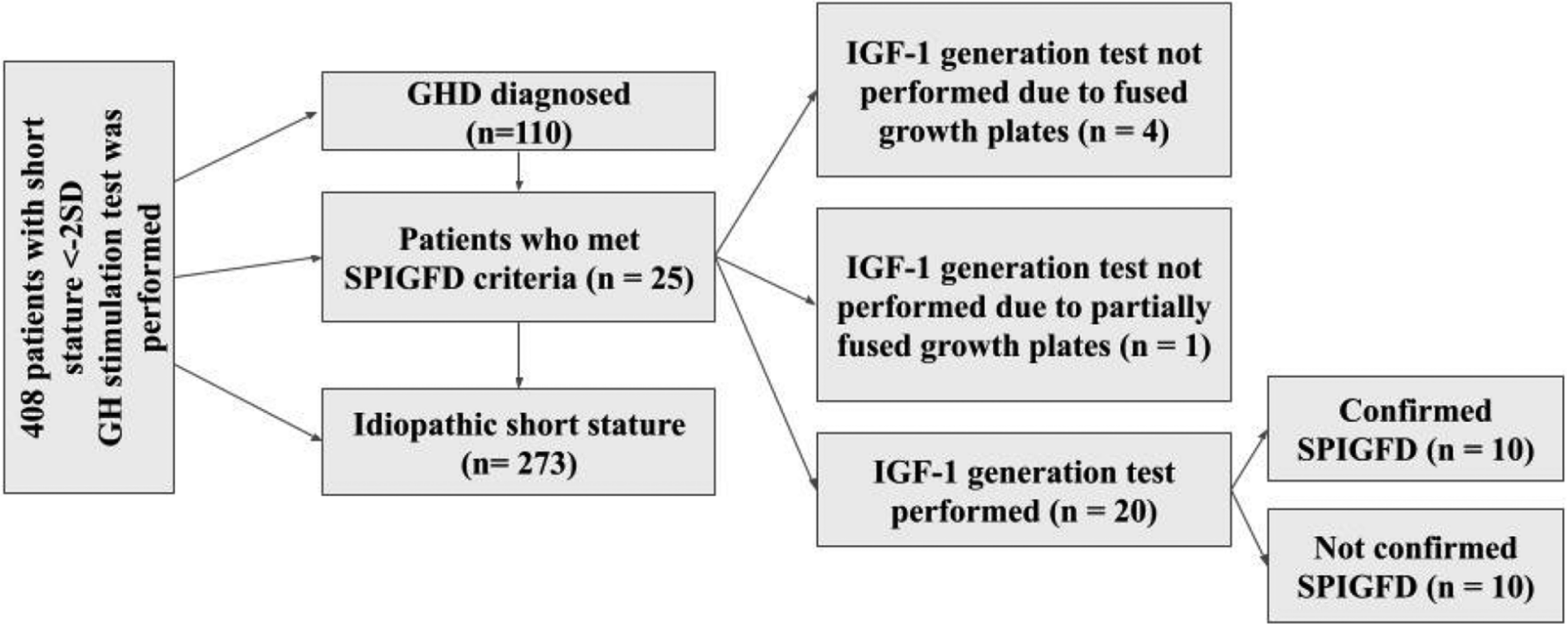
Selection of study patients from 2012 to 2023 years. GHD, growth hormone deficiency; SPIGFD, severe primary IGF-1 deficiency.

A retrospective study was conducted on patients with severe primary IGF-1 deficiency treated with rIGF-1 (*mecasermin*) at the Endocrinology Clinic of LSMUL Kaunas Clinics from 2017 to 2024. However, the search for patients meeting SPIGFD criteria was applied from 2012. Patients were diagnosed with severe primary IGF-1 deficiency if their height was extremely low, defined as less than −3.0 standard deviations (SD) from the mean for their sex and age, and their IGF-1 concentration was below the 2.5th percentile (or <−2 SD) for their sex and age, while their growth hormone (GH) concentration was normal (confirmed by GH stimulation tests). Normal GH concentration is considered then GH peak is ≥10 ng/ml during GH stimulation tests. An IGF-1 generation test was performed on all patients to evaluate GH sensitivity. The standard test involved a four-day procedure (Figure 1) (12, 13). Baseline IGF-1 concentration was determined, and then daily rhGH was injected subcutaneously in the evening for four consecutive days at a daily dose of 33 μg/kg body weight. The IGF-1 response was assessed by measuring IGF-1 concentration the next morning at 9 a.m., 12–15 h after the fourth rhGH injection. An increase of IGF-1 concentration less than 50% confirmed the diagnosis of severe primary IGF-1 deficiency. Data from a total of 10 patients (7 boys and 3 girls) were selected and analysed (Figure 2). Anthropometric, hormonal, and metabolic indicators were evaluated.

### Data collection

The following data were collected from the outpatient health records of the patients: parents’ heights, gestational age in weeks, birth weight/height, height/weight at the start of rhIGF-1 treatment, height/weight at each visit until the end of rhIGF-1 treatment, bone age (BA) assessed before the treatment and on the most recent x-ray of the non-dominant hand, and body mass index (BMI) calculated at each visit. The standard deviations for these measurements according to age and sex were derived based on the National Growth Charts (0–18 years) (14). The mean treatment duration was 4.44 years, with a range of 2.49–6.91 years (5th–95th percentile).

### General data of study subjects

The average age at the start of rhIGF-1 (mecasermin) treatment was 9.66 years (boys - 9.68 years, girls - 9.63 years); the youngest patient was 3.24 years old, and the oldest was 12.86 years old. The average height SDS (standard deviation score) at the start of treatment was −3.69 SDS. At the beginning of treatment, nine patients were at Tanner stage 1 of sexual development, and one patient was at Tanner stage 2. The characteristics of the study group are presented in Table 1. The initial dose of mecasermin was 40 μg/kg twice daily, which is the recommended starting dose. Subsequently, the dosage was gradually increased according to recommendations, with the highest dose used being 120 μg/kg body weight twice daily. All children were given the same adherence recommendations as provided by the drug supplier. The treatment monitoring was applied uniformly. Each patient was provided with a personal glucometer (Accu-Check Performa) for self-monitoring of blood glucose (SMBG). They were instructed to check their blood glucose levels 2 h after the rhIGF-1 injection and whenever they experienced symptoms indicative of hypoglycemia, such as sweating, dizziness, or weakness. Hypoglycemia was defined as any occurrence of typical clinical symptoms, confirmed by SMBG or laboratory-tested venous blood with glucose levels below 3.9 mmol/L. Severe hypoglycemic episodes were classified as those with glucose levels below 2.8 mmol/L, requiring hospitalization or assistance from another person. These readings were recorded in patient diaries and reviewed during follow-up visits. Follow-up visits were conducted every three months. During each follow-up visit, IGF-1 and fasting glucose concentrations were assessed. Every six months, thyroid-stimulating hormone (TSH), free thyroxine (FT4), and serum electrolyte concentrations were evaluated. Every 12 months, a cardiologist consultation with echocardiography and an otolaryngologist consultation with audiometry were performed to monitor for potential lymphoid organ hypertrophy due to rhIGF-1 treatment, and bone age (BA) was assessed.

**Table 1:** Characteristics of study patients before starting rIGF-1 treatment.

Characteristic	Mean (*n* = 10)	Min	1st Quartile	Median	3rd Quartile	Max
Height (SDS)	−3.69	−5.48	−4.17	−3.40	−3.06	−2.93
Weight (SDS)	−3.30	−5.37	−4.02	−3.46	−2.60	−1.53
BMI (SDS)	−1.11	−2.29	−1.90	−1.18	−0.51	0.28
GH peak (mU/L)	39.32	23.3	32.6	33.3	51.5	55.9
Basal IGF-1 (nmol/L)	12.84	8.3	10.9	12.2	12.2	20.7
Prolactin (mU/L)	219.2	116	239	247	250	272
Fasting glucose (mmol/L)	4.40	3.56	3.84	4.32	4.59	4.86
Fasting insulin (mU/L)	5.37	3	5.1	5.1	5.3	6
HOMA-IR	1.05	0.47	0.87	1.02	1.04	1.3
ALP (U/L)	150.5	127	148	159	168	174
Cholesterol (mmol/L)	4.41	3.63	3.81	4.81	4.87	5.4
HDL (mmol/L)	1.42	1.15	1.22	1.37	1.41	1.91
LDL (mmol/L)	2.69	1.9	2.25	3.28	3.33	3.46
Triglycerides (mmol/L)	0.69	0.37	0.38	0.67	0.76	0.96

SDS, standard deviation score; BMI, body mass index; GH, growth hormone; HOMA-IR: homeostatic model assessment for insulin resistance; ALP, alkaline phosphatase; HDL, high-density lipoprotein cholesterol; LDL, low-density lipoprotein cholesterol.

### Statistical analysis

Descriptive and analytical statistical methods were used. To compare the means of the same quantitative variable in two groups, when the distribution of the variable met the normal distribution, the Student's *t*-test was used. For comparing two paired samples that did not meet the normality condition, the non-parametric Wilcoxon signed-rank test was employed.

The data for parametric variables are presented as mean and standard deviation (SD), while the data for non-parametric variables (since the sample size is <30) are presented as median with the 1st and 3rd quartiles. To evaluate the association between qualitative variables, the chi-square (χ^2^) test was applied. Differences were considered statistically significant when *p* < 0.05. The frequency and type of side effects were reported as percentages.

### Bioethics

The study was approved by Kaunas Regional Ethics Committee of Biomedical Research (No BEC-MF-207, approved 23 February 2023). All procedures were carried out with adequate understanding and written consent of the participants and their parents or caregivers were obtained as well. The investigation was carried out in accordance with the Declaration of Helsinki.

## Results

### Growth outcomes

**Figure 3:**
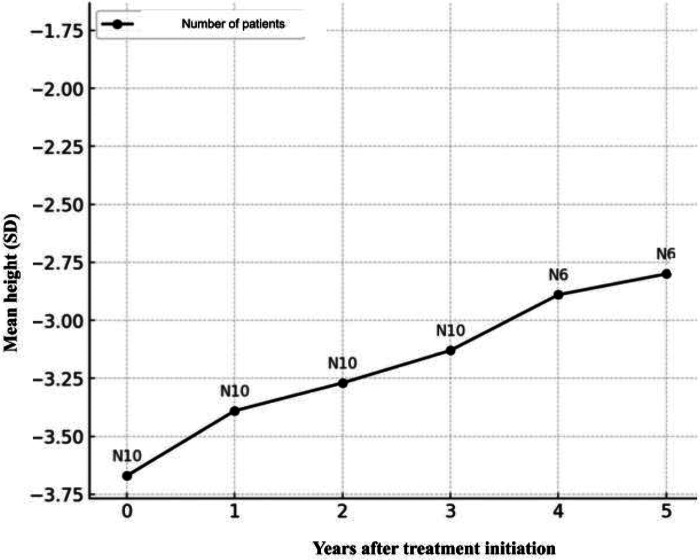
Average change in height (SD) per year from the start of rhIGF-1 treatment.

The height of the entire study group improved with mecasermin treatment. The average height before treatment was −3.69 ± 0.82 SDS. Upon completion of treatment or at the last visit for patients still undergoing treatment, the average height increased to −2.93 ± 1.26 SDS, *p* < 0.01. The average height SDS increase for all patients was 0.76 ± 0.64 (1st quartile 0.29 SDS, 3rd quartile 1.14 SDS) (Figure 3).

Patient No. 4 was confirmed to have a Noonan syndrome-like disorder with a pathogenic mutation in the *SHOC2* gene, and patient No. 8 was confirmed to have mitochondrial disease (a possibly pathogenic variant m.3761C>A in the MT-ND1 gene causing mitochondrial complex I deficiency). To compare if these genotypes resulted in a poorer response to treatment, the height SDS change for patients 4 and 8 was calculated to be 0.10 ± 0.21, while the average change in the other patient group was 0.88 ± 0.64. This shows a statistically significant difference in response to treatment between these two patients and the other group members, as confirmed by *t*-test results (*p* = 0.03).

**Figure 4:**
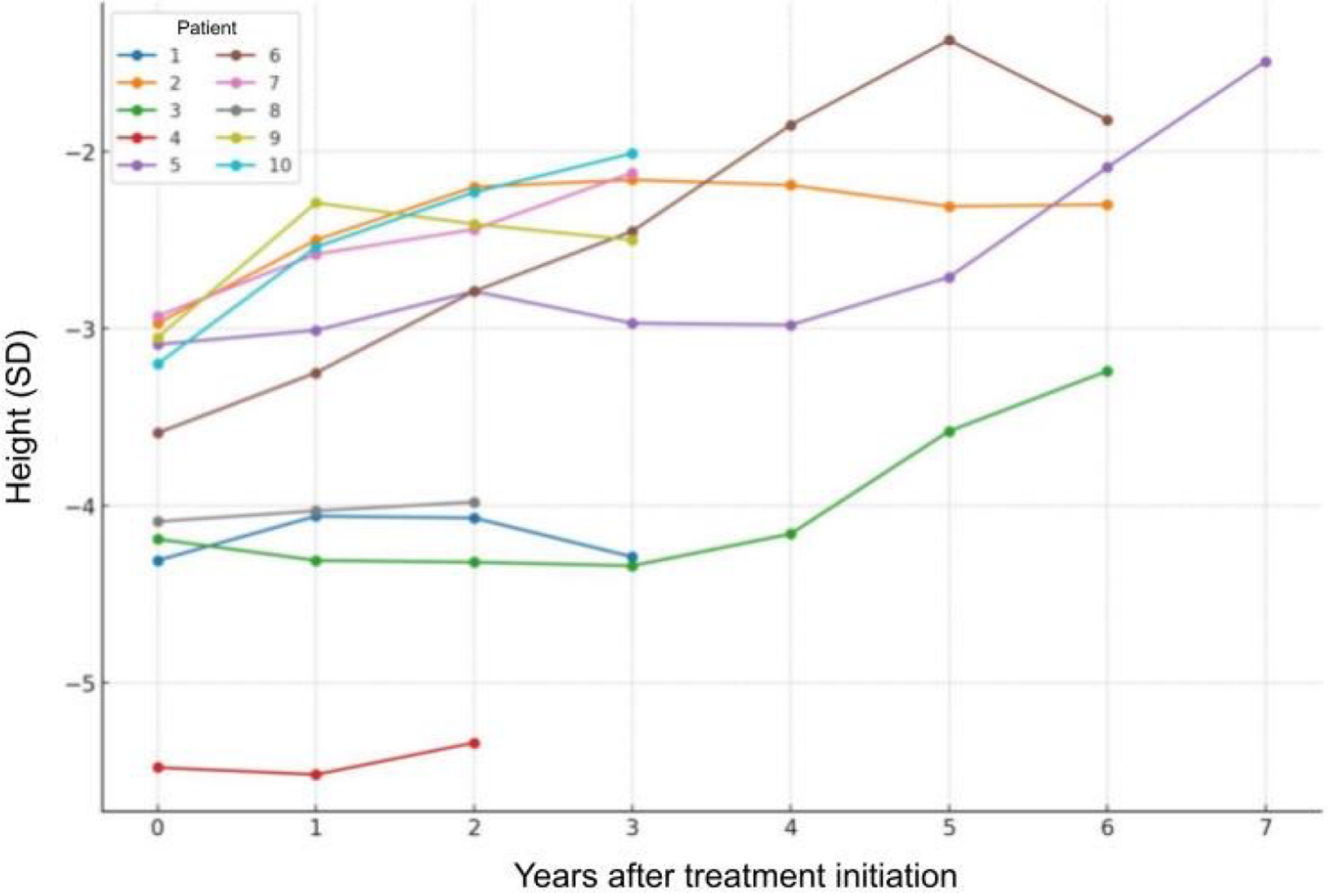
Individual growth trajectories of each patient from the start of rhIGF-1 treatment.

Figure 4 is presented to visually show the individual growth trajectories of each patient from the start of treatment, highlighting the varied response to recombinant IGF-1 and emphasizing the importance of individualized treatment adjustments based on specific patient outcomes.

### Weight outcomes

**Figure 5:**
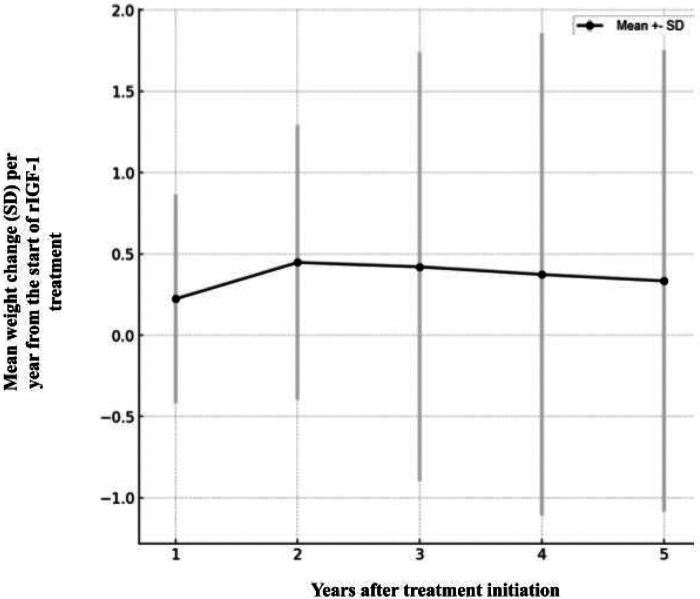
Average change in body mass (SD) per year from the start of rIGF treatment.

During the treatment period, the average body mass SDS of the patients increased by 0.37 ± 1.35 (1st quartile −0.33 SDS, 3rd quartile 0.92 SDS). This indicates that, on average, body mass SDS increased, but there were significant differences in BMI among the patients. The change in body mass SDS varied greatly between individual patients: some patients’ body mass SDS increased, while others decreased. For example, one patient's body weight SDS increased by 0.66 over 1,197 treatment days, while another patient's body weight SDS decreased by −1.02 over 793 days. This suggests that the observed average change in body mass SDS could have occurred by chance, and no statistically significant relationship between treatment duration and change in body mass SDS was observed (Figure 5).

### Side effects

Side effects of the treatment occurred in five patients (50%). Among the patients who experienced hypoglycemia, three (30%) had mild episodes characterized by weakness, which were promptly corrected with fast-acting carbohydrates. For one of them, hypoglycemia episodes were associated with physical exercise. However, one patient had a single hypoglycemic episode, classified as severe due to a glucose level below 2.8 mmol/L, but the patient only exhibited mild symptoms such as weakness and sweating. This episode was detected during a routine check, and the patient was hospitalized for monitoring, though no further complications occurred. All patients experienced only a single episode of hypoglycemia throughout the study. One patient (10%) had hyperlipodystrophic changes at the injection sites, and the same patient experienced lip and facial swelling as well as headaches. Hypertrophy of the lymphoid tissue of the pharyngeal tonsils occurred in one patient (10%). One patient (10%) had episodes of hyperglycemia up to 10 mmol/L, but no other symptoms of diabetes were observed. One female patient developed hirsutism (Farriman-Gallway score – 10) (15) after six months of treatment. However, the relationship between this symptom and the treatment remained unclear.

## Discussion

Our study demonstrated a consistent improvement in height SD among the treated patient group. After one year of treatment with mecasermin, the average height SD in our study group increased from −3.69 to −2.93, showing significant improvement compared to the baseline. This result is similar to those obtained in other studies, although some studies show greater variability. For instance, a study conducted in the USA (6) found that the average height change at the final analysis stage was +1.9 SD, ranging from +0.1 to +4.7 SD. In this study, nine patients improved their height by ≥ +2.0 SD, while twelve patients achieved only up to +1.7 SDS height increase, indicating an individual response to rhIGF-1 treatment (6, 11, 13).

A study conducted in Poland (12) shows that after 36 months of mecasermin treatment, the average height SD improved from −3.52 ± 0.82 to −2.25 ± 0.91, indicating a significant increase in height SD during the treatment period (*p* < 0.01). The average height gain was 1.45 ± 1.06 SD, demonstrating a stable and long-term treatment effect.

The results of the European Increlex® Growth Forum database also provide important insights. The main findings of the study show that treatment significantly improved height SD until the pubertal period, compared to the initial measurement. For example, among patients who reached the end of puberty, the average height SD increased from −3.7 to −2.6 in boys and from −3.1 to −2.3 in girls. This study showed a delay of about 1.5 years despite continuous rhIGF-1 treatment, and the growth spurt rate during puberty occurred later and was slightly lower than that of healthy control group children (16).

In five clinical studies with rhIGF-1 (Increlex®), significant height improvement was observed in children with SPIGFT. The initial height SDS of these patients was −6.9 ± 1.8. After the first year of mecasermin treatment, the height SDS improved to −6.1 ± 1.8, and continued to improve in subsequent years, reaching −5.6 ± 1.7, −5.3 ± 1.7, −5.1 ± 1.7, −5.0 ± 1.7, and finally stabilized around −4.9 ± 1.6. These data confirm the long-term efficacy of mecasermin therapy, consistently improving height indices over the treatment period. These results show that mecasermin effectively improves height SD in the long term, especially for patients who start treatment early in childhood (11).

The review of these studies shows that early and continuous mecasermin treatment can be very effective in improving height SD in children with SPIGFT, although treatment outcomes can vary greatly depending on the patient's baseline condition and the timing of treatment initiation (1, 6, 12).

The individual variability in treatment outcomes observed in our study indicates that genetic, clinical, and demographic factors significantly influence patients’ response to mecasermin treatment. The impact of genotypes on therapeutic response was particularly important. During our study, patients with Noonan-like syndrome (*SHOC2* gene mutation), mitochondrial pathology, and *EXT2* gene variant showed distinctive treatment responses. For example, patients with Noonan syndrome (*SHOC2* mutation) had a lower growth rate increase with rhIGF-1 treatment compared to other subjects with standard GH receptor gene deletions. Studies on patients with Noonan syndrome, who have pathogenic mutations affecting the RAS/MAPK pathway, have shown that genetic differences significantly influence treatment outcomes, confirming the impact of genotype on therapeutic response (17, 18).

Additionally, the pathogenic variant of the *EXT2* gene, associated with hereditary multiple osteochondromas syndrome, led to unpredictable growth changes despite the applied therapy (19). Moreover, mitochondrial pathology, which improves due to IGF-1 treatment, affected overall body energy production and metabolism, potentially modifying and reducing the effectiveness of mecasermin treatment (19). Such genetic features not only explain the clinical response variability to treatment but also highlight the need to individualize treatment strategies based on each patient's genotype. These insights emphasize the importance of personalized medicine, indicating that future clinical practices should apply individualized treatment strategies tailored to specific genetic profiles and associated pathologies (20–23).

IGF-1 (mecasermin) and GH treatments both enhance growth in children with deficiencies, but they operate through different mechanisms and have distinct efficacy and side effect profiles. IGF-1 directly supplements IGF-1 levels, bypassing GH stimulation, and is particularly effective for those with GH insensitivity or receptor mutations. In contrast, GH therapy boosts growth by increasing IGF-1 levels and is effective for a wider range of growth issues, including GH deficiency and idiopathic short stature, while also improving body composition and metabolic profiles (23).

Studies show that IGF-1 treatment initially results in significant growth, averaging 8.0–8.5 cm annually in the first year, but its effectiveness declines in subsequent years, often dropping below 6.0 cm in the second year. In comparison, children with GH deficiency can achieve growth rates of 9–14 cm per year with GH therapy. Despite these benefits, GH can cause side effects like edema and insulin resistance, while IGF-1 may lead to hypoglycemia. Long-term rhIGF-1 therapy is limited in achieving normal height in severe cases due to its negative impact on endogenous GH levels. These findings suggest that combined therapies with both rhIGF-1 and GH may improve growth outcomes more effectively than monotherapy, emphasizing the need for personalized treatment strategies based on individual genetic factors and responses (23).

Hypoglycemia is one of the most commonly reported adverse events associated with rhIGF-1 therapy. In our study, hypoglycemia was observed in several patients, with most episodes being mild and manageable with fast-acting carbohydrates. These findings align with previous research, such as data from the European Increlex® Growth Forum Database (Eu-IGFD), which also highlighted hypoglycemia as a frequent adverse event, particularly in younger patients or those with a prior history of hypoglycemia. Given that continuous glucose monitoring (CGM) was not utilized in this study, some hypoglycemic episodes, especially asymptomatic ones, may have gone undetected. Future studies should consider incorporating CGM to provide a more comprehensive assessment of glycemic fluctuations in children undergoing rhIGF-1 therapy. Additionally, based on our findings, it is advisable for clinicians to evaluate the sufficiency of current glucose monitoring protocols, particularly during periods of increased physical activity or illness. The occurrence of hypoglycemia underscores the importance of careful glucose monitoring and patient education to reduce risks during rhIGF-1 therapy. While most hypoglycemic events are mild, the potential for severe episodes necessitates vigilance, particularly in patients with additional risk factors such as Laron syndrome (24).

## Conclusions

This study involving patients with SPIGF-1 deficiency treated with rhIGF-1 analog demonstrated that rhIGF-1 is an effective growth-promoting agent, especially in the first year of treatment. However, the long-term perspective shows a decrease in growth rate, necessitating continuous monitoring and treatment adjustments.

The study revealed significant aspects related to treatment initiation and side effects. Side effects like hypoglycemia required adjustments in treatment strategies, considering patients’ age and prior health conditions. Optimal treatment efficacy is achieved through comprehensive patient monitoring and individualized dosing, emphasizing a personalized approach based on specific health characteristics and treatment response dynamics.

Further research with larger patient samples and longer follow-up is recommended to optimize treatment protocols and ensure maximum benefit. This thesis contributes to the scientific advancement in treating rare growth disorders and highlights the importance of improving clinical protocols for safety and efficacy.

## Study limitations

This study has several limitations. SPIGFD is very rare condition and due to the low prevalence of the disease, the patient cohort was relatively small. This limited sample size may affect the generalisability of the study findings. Moreover, genetic analysis was not performed for all patients in our patients’ group. The absence of genetic data may have impacted the ability to predict outcomes of targeted treatment. In addition, glycemia was not consistently observed using a continuous glucose monitoring (CGM) system. Utilizing CGM could have provided more detailed and accurate data on glycemic fluctuations and potential hypoglycemic episodes, leading to a better understanding of the side effects associated with the treatment.

## Data Availability

The raw data supporting the conclusions of this article will be made available by the authors, without undue reservation.
